# Pharmacogenetics of cytochrome P450 2B6 (CYP2B6): advances on polymorphisms, mechanisms, and clinical relevance

**DOI:** 10.3389/fgene.2013.00024

**Published:** 2013-03-05

**Authors:** Ulrich M. Zanger, Kathrin Klein

**Affiliations:** ^1^Dr. Margarete Fischer-Bosch Institute of Clinical PharmacologyStuttgart, Germany; ^2^The University of TuebingenTuebingen, Germany

**Keywords:** bupropion, cyclophosphamide, cytochrome P450, drug metabolism, drug–drug interaction, efavirenz, pharmacogenetics, pharmacogenomics

## Abstract

Cytochrome P450 2B6 (CYP2B6) belongs to the minor drug metabolizing P450s in human liver. Expression is highly variable both between individuals and within individuals, owing to non-genetic factors, genetic polymorphisms, inducibility, and irreversible inhibition by many compounds. Drugs metabolized mainly by CYP2B6 include artemisinin, bupropion, cyclophosphamide, efavirenz, ketamine, and methadone. *CYP2B6* is one of the most polymorphic CYP genes in humans and variants have been shown to affect transcriptional regulation, splicing, mRNA and protein expression, and catalytic activity. Some variants appear to affect several functional levels simultaneously, thus, combined in haplotypes, leading to complex interactions between substrate-dependent and -independent mechanisms. The most common functionally deficient allele is CYP2B6*6 [Q172H, K262R], which occurs at frequencies of 15 to over 60% in different populations. The allele leads to lower expression in liver due to erroneous splicing. Recent investigations suggest that the amino acid changes contribute complex substrate-dependent effects at the activity level, although data from recombinant systems used by different researchers are not well in agreement with each other. Another important variant, CYP2B6*18 [I328T], occurs predominantly in Africans (4–12%) and does not express functional protein. A large number of uncharacterized variants are currently emerging from different ethnicities in the course of the 1000 Genomes Project. The *CYP2B6* polymorphism is clinically relevant for HIV-infected patients treated with the reverse transcriptase inhibitor efavirenz, but it is increasingly being recognized for other drug substrates. This review summarizes recent advances on the functional and clinical significance of CYP2B6 and its genetic polymorphism, with particular emphasis on the comparison of kinetic data obtained with different substrates for variants expressed in different recombinant expression systems.

## INTRODUCTION

**FIGURE 1:**
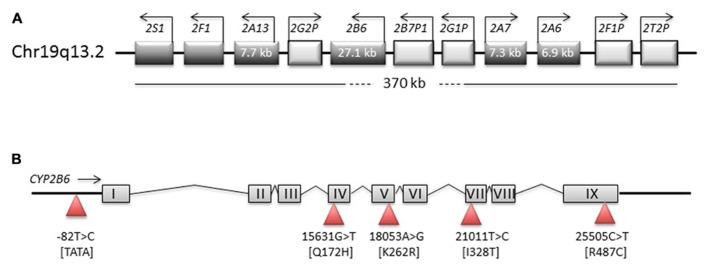
**Structure of genomic regions on chromosome 19**. Schematic representation of the *CYP2ABFGST-*cluster on chromosome 19 **(A)** and of the *CYP2B6* gene **(B)**. Boxes in the **(A)** represent genes, with direction of transcription indicated by arrows. The **(B)** represents CYP2B6 exons and introns and shows the most important SNPs as triangles.

The cytochrome P450 (CYP) enzyme CYP2B6 is one of about a dozen human CYPs that are primarily involved in the biotransformation of drugs and other xenobiotics. The *CYP2B6* gene and its closely related pseudogene, *CYP2B7*, are located in a tandem head-to-tail arrangement within a large *CYP2* gene cluster on the long arm of chromosome 19 (Hoffman et al., 2001; **Figure 1**). The orthologous genes in dog, mouse, and rat are termed *CYP2B11*, *Cyp2b10*, and *CYP2B1*, respectively, but in contrast to other mammalian species, CYP2B6 is the only functional isozyme of its subfamily in humans (Nelson et al., 2004). Owing to the existence of extensive genetic polymorphism as well as strong inhibitors and inducers, its activity is highly variable in the population. For some clinically used drugs including the antiretroviral agents efavirenz and nevirapine, *CYP2B6 *single nucleotide polymorphisms have been shown to be useful predictors of pharmacokinetics and drug response (reviewed in Zanger et al., 2007; Telenti and Zanger, 2008; Rakhmanina and van den Anker, 2010). However, recent data indicate that pharmacogenetic mechanisms are complex, appear on several levels of gene expression from the initial mRNA transcript to splice variants (pre-mRNA splicing and mRNA expression) to altered proteins, and affect function in various ways including substrate-dependent and substrate-independent effects. Several previous reviews are available that cover the biochemical pharmacology, molecular genetics, and pharmacogenetics of this enzyme at various degrees of detail (Ekins and Wrighton, 1999; Turpeinen et al., 2006; Hodgson and Rose, 2007; Zanger et al., 2007; Wang and Tompkins, 2008; Mo et al., 2009; Turpeinen and Zanger, 2012). The purpose of this review is to summarize recent advances in areas that have an impact on variable expression of CYP2B6 and the mechanisms and impact of *CYP2B6 *polymorphism, as observed by various *in vitro *approaches as well as in *in vivo *studies, and to discuss their functional and clinical implications.

## VARIABILITY OF EXPRESSION AND TRANSCRIPTIONAL REGULATION

Cytochrome P450 2B6 is primarily expressed in the liver where its contribution to the total microsomal P450 pool has been estimated to be within a range of about 1–10%, with a large inter-individual variability at protein level of roughly 100-fold (see Zanger et al., 2007 for review and references therein). Although some earlier studies reported expression in only a fraction of human livers, newer studies with better antibodies found CYP2B6 to be present in all investigated human adult liver samples (Hofmann et al., 2008) while up to one-third of pediatric samples contained no detectable protein (Croom et al., 2009). In the latter study, ontogenic differences were studied in liver microsomes from 217 pediatric liver donors. Hepatic median CYP2B6 protein levels were about twofold higher in the period between birth and 30 days postnatal compared to fetal samples, and protein levels varied already over 25-fold in both of these age groups (Croom et al., 2009). Maturation effects may further depend on genotype, as suggested in a study on HIV-infected children treated with efavirenz (Sueyoshi et al., 1999; Wang et al., 2003; Faucette et al., 2004, 2007).

One of the most important factors contributing to intra- as well as inter-individual variability is enzyme induction, i.e., *de novo* protein synthesis following exposure to certain chemicals. Regulation of *CYP2B *gene expression represents the archetypal example of enzyme induction (Remmer et al., 1973). Human CYP2B6 is strongly inducible by several drugs including “classical” inducers such as rifampicin, phenytoin, and phenobarbital involving a so-called phenobarbital-responsive enhancer module (PBREM) at -1.7 kb of the *CYP2B6 *gene promoter, and a distal xenobiotics-responsive enhancer module (XREM, -8.5 kb), to which pregnane X receptor (PXR, *NR1I2*) and/or constitutive androstane receptor (CAR, *NR1I3*) bind to mediate increased transcription (Sueyoshi et al., 1999; Wang et al., 2003; Faucette et al., 2004, 2007). Since other CYPs are regulated by overlapping sets of nuclear receptors, CYP2B6 is often co-induced with CYP2C enzymes and CYP3A4. CYP2B6 inducers identified to date include cyclophosphamide (Gervot et al., 1999), hyperforin (Goodwin et al., 2001), artemisinin antimalarials (Simonsson et al., 2003; Burk et al., 2005), carbamazepine (Oscarson et al., 2006; Desta et al., 2007), metamizole (Saussele et al., 2007; Qin et al., 2012), ritonavir (Kharasch et al., 2008), the insect repellent *N*,*N*-diethyl-*m*-toluamide (DEET; Das et al., 2008), statins (Feidt et al., 2010), efavirenz (Ngaimisi et al., 2010; Habtewold et al., 2011). Interestingly, in the latter study, gender influenced the inducibility of efavirenz 8-hydroxylation, which was higher in women than in the men (Ngaimisi et al., 2010). In addition to therapeutic drugs, pesticides were found to be powerful inducers of CYP2B6 and other CYPs through interaction with both PXR and CAR (Das et al., 2008). Induction of CYP2B6 and other cytochromes P450 and its clinical consequences has been reviewed by others (Pelkonen et al., 2008; Mo et al., 2009).

Sex differences in liver expression have been observed in a number of studies. Females liver donors had higher amounts of CYP2B6 mRNA (3.9-fold), protein (1.7-fold), and enzyme activity (1.6-fold) compared to male subjects in a study of 80 ethnically mixed samples (Lamba et al., 2003). In a study with 235 Caucasian liver donors, female samples had 1.6-fold higher expression level of CYP2B6 mRNA, however, this difference did not translate into higher protein and activity levels and no sex difference was found when only liver donors without presurgical drug exposure were considered (Hofmann et al., 2008). Discrepant effects of sex on pharmacokinetics of CYP2B6 substrates, which may be due to other confounders such as age or smoking status, were also found *in vivo*. Higher bupropion hydroxylation rates were found in adolescent females compared to males (Stewart et al., 2001) but not in adults (Hsyu et al., 1997). For efavirenz, several studies reported elevated plasma concentrations in female compared to male patients, which is in contrast to the above-mentioned *in vitro* findings and may be explained by other factors such as differences in body fat content and distribution (Burger et al., 2006; Nyakutira et al., 2008; Mukonzo et al., 2009). The influence of age on CYP2B6 expression may also depend on sex, as only males showed a significant increase of liver CYP2B6 at higher age (Yang et al., 2010).

Besides liver, CYP2B6 is also consistently expressed in different parts of respiratory and gastrointestinal tracts, including lung and nasal mucosa, and also in skin and the kidneys (Choudhary et al., 2003; Dutheil et al., 2008; Thelen and Dressman, 2009; Leclerc et al., 2010). The significance of CYP2B6 in these extrahepatic tissues is currently unknown, but it should be remembered that the enzyme is probably the most important one for many environmental toxins such as pesticides, and its presence in tissues with barrier function may thus contribute substantially to protection against these chemicals. In addition, the presence of CYP2B6 in brain has been demonstrated in human and primate brain tissue samples and smoking, alcohol consumption, and genetic polymorphism have been suggested to contribute to its variability in this organ (Miksys et al., 2003). In general, CYP levels in extrahepatic tissues are far below those of liver, but the localization to specific regions in the brain may contribute to the activation or inactivation of centrally acting drugs and to neurological side effects of certain medications or abused drugs, e.g., “ecstasy” [1-methyl-4-phenyl-1,2,3,6-tetrahydropyridine (MPTP), see below]. This may also explain why efficacy for some centrally acting drugs is not well correlated to their plasma levels. The potential role of brain-expressed CYPs including CYP2B6 in the biotransformation of centrally acting drugs has been reviewed by others (Meyer et al., 2007; Ferguson and Tyndale, 2011).

## THE CHEMICAL INTERACTION PROFILE OF CYP2B6

Recent studies have revealed crystal structures of the CYP2B6 wild-type and K262R variant in complex with various inhibitors at providing first views into its active site and its plasticity to adopt different conformations when binding different ligands (Gay et al., 2010; Shah et al., 2011; Wilderman and Halpert, 2012). Substrates of CYP2B6 are usually fairly lipophilic, neutral or weakly basic non-planar molecules with one or two hydrogen bond acceptors (Lewis et al., 1999, 2004). The CYP2B6 substrate selectivity comprises many diverse chemicals, including not only clinically used drugs but also many environmental chemicals such as pesticides (Turpeinen et al., 2006; Hodgson and Rose, 2007; Turpeinen and Zanger, 2012). Therapeutically important drugs metabolized primarily by CYP2B6 include the prodrug cyclophosphamide, which is converted to the direct precursor of the cytotoxic metabolites, phosphoramide mustard and acrolein, by 4-hydroxylation (Huang et al., 2000; Roy et al., 2005), the non-nucleoside reverse transcriptase inhibitor (NNRTI), efavirenz, which is 8-hydroxylated to become pharmacologically inactive (Ward et al., 2003; Desta et al., 2007), the atypical antidepressant and smoking cessation agent bupropion, which is converted to pharmacologically active hydroxybupropion (Faucette et al., 2000; Hesse et al., 2000; Turpeinen et al., 2005b), the anesthetics propofol (Court et al., 2001; Oda et al., 2001) and ketamine (Desta et al., 2012), the analgesic pethidine (meperidine; Ramírez et al., 2004); the μ-opioid receptor agonist, methadone (Totah et al., 2008), the antimalarial artemisinin (Svensson and Ashton, 1999; Asimus and Ashton, 2009), among numerous additional metabolic pathways of other drugs, to which CYP2B6 contributes in part, such as the antiretroviral, nevirapine (Erickson et al., 1999), and many others (Turpeinen and Zanger, 2012). Metabolic pathways suitable as probe for CYP2B6 activity include S-mephenytoin *N*-demethylation (Ko et al., 1998), bupropion hydroxylation (Faucette et al., 2000; Fuhr et al., 2007) and efavirenz, based on *in vitro* investigations (Ward et al., 2003; Desta et al., 2007).

Endogenous substances metabolized by the enzyme include arachidonic acid, lauric acid, 17beta-estradiol, estrone, ethinylestradiol, and testosterone 16α- and 16β-hydroxylation (Ekins et al., 1998).

Cytochrome P450 2B6 furthermore participates in the biotransformation of the abused drug “ecstasy” (*N-*methyl-3,4-methylenedioxymethamphetamine, MDMA), which is *N-*demethylated leading to potentially neurotoxic metabolites (Kreth et al., 2000). It also plays a minor role in nicotine metabolism (Yamazaki et al., 1999; Yamanaka et al., 2005). CYP2B6 has furthermore been found to be of importance in the metabolism of pesticides and other environmental chemicals and pollutants (Hodgson and Rose, 2007). In particular the bioactivating oxidation of the organophosphorus insecticides chlorpyrifos (Crane et al., 2012) and methyl parathion (Ellison et al., 2012a) to their more toxic oxon metabolites is mainly catalyzed by CYP2B6, a public health concern due to their worldwide use and documented human exposures (Ellison et al., 2012b). Further environmental substrates are the insecticide and endocrine disruptor methoxychlor, the extensively used insect repellent *N*,*N*-diethyl-*m*-toluamide (Das et al., 2008), profenofos and other pesticides (Abass and Pelkonen, 2012), as well as the tobacco-specific nitrosamine, 4-(methylnitrosamino)-1-(3-pyridyl)-1-butanone (NNK; Smith et al., 2003), aflatoxin B1 (Code et al., 1997), and others (Hodgson and Rose, 2007; Abass and Pelkonen, 2012).

Several structurally unrelated drugs have been shown to inhibit CYP2B6 and many of them do that in a mechanism-based, irreversible manner (Turpeinen et al., 2006; Turpeinen and Zanger, 2012). The thienopyridine derivatives clopidogrel and ticlopidine are prodrugs that selectively inhibit platelet aggregation and have been in clinical use for the prevention of atherothrombotic events for several years. Both of them are potent mechanism-based inhibitors of CYP2B6 (Richter et al., 2004; Zhang et al., 2011a). The established anticancer agent, thioTEPA (*N*,*N*′,*N*′′-triethylenethiophosphoramide) was also found to be a highly selective and mechanism-based CYP2B6 inhibitor (Rae et al., 2002; Harleton et al., 2004; Richter et al., 2005). A comparison of several selective inhibitors revealed that 2-phenyl-2-(1-piperidinyl)propane is probably the most selective CYP2B6 inhibitor *in vitro* (Walsky and Obach, 2007). Recent *in vitro *observations identified the progesterone receptor antagonist, mifepristone (RU486; Lin et al., 2009); the anti-Parkinsonian agent selegiline (the R-enantiomer of deprenyl; Sridar et al., 2012), methadone (Amunugama et al., 2012), and tamoxifen (Sridar et al., 2012) as potent mechanism-based inhibitors. *In vivo *drug–drug interactions have been reported, for example, between thioTEPA and cyclophosphamide (Huitema et al., 2000), clopidogrel and bupropion (Turpeinen et al., 2005a), voriconazole and efavirenz (Liu et al., 2008; Jeong et al., 2009), clopidogrel and efavirenz (Jiang et al., 2012), and between ticlopidine and ketamine (Peltoniemi et al., 2011). Furthermore, certain non-pharmaceutical compounds like particular benzylpyridine derivatives have been characterized as very potent inhibitors of CYP2B6 (Korhonen et al., 2007) and have been utilized for structural modeling experiments (Gay et al., 2010).

## PHARMACOGENETICS OF *CYP2B6*

The *CYPalleles* website^1^ currently lists 37 distinct star-alleles, i.e., gene haplotypes with a distinct variant amino acid sequence or with demonstrated functional effect (last accessed: February 21st, 2013). More than 30 amino acid-changing single-nucleotide polymorphisms (SNPs) occur in different combinations and together with additional non-coding variants and many more SNPs not yet assigned to particular haplotypes. The worldwide variations in SNP frequencies have been reviewed recently (Li et al., 2012). **Table 1** lists the most important variants in terms of frequency and functional impact and summarizes updated structural, functional, and frequency information for different ethnicities. In addition to the *CYPallele* website, further valuable information about *CYP2B6* SNPs and pharmacogenetics are available on the websites of *The Pharmacogenomics Knowledgebase*^2^, the *NCBI portal for short genetic variations*, dbSNP^3^, the *1000 Genomes Catalog of Human Genetic Variation*^4^, as well as the NHLBI exome sequencing project^5^.

**Table 1:** Summary data on selected genetic polymorphisms of *CYP2B6*.

CYP allele designation^a^	Key mutation(s)^b^ rs number	Location, protein effect	Allele frequencies^c^	Functional effect
CYP2B6*4	g.18053(c.516) A>G rs2279343	K262R (isolated)	0.00 AA, Af 0.04 Ca 0.05–0.12 As	↑ Expression, moderate substrate-dependent effects
CYP2B6*5	g.25505(c.1459) C>T rs3211371	R487C	0.01–0.04AA, Af 0.09–0.12 Ca 0.05–0.12 Hs 0.01–0.04 As	↓ Expression, in part compensated by ↑ specific activity
CYP2B6*6	g.15631(c.516) G>T rs3745274 and g.18053(c.785)A>G rs2279343	Q172H K262R	0.33–0.5 AA, Af 0.10–0.21 As 0.14–0.27 Ca 0.62 PNG	↓ Expression; ↓activity with efavirenz *in vivo*; some other substrates show ↑ activity
CYP2B6*18	g.21011(c.983)T>C rs28399499	I328T	0.04–0.08 AA 0.05–0.12, Af 0.01 HS 0.00 As, Ca, PNG	↓ Expression and activity
CYP2B6*22	g.-82T>C rs34223104	promoter (TATA-box)	0.00–0.025 AA, Af, As 0.024 Ca, Hs	↑ Expression and activity ↑ Inducibility *in vitro*

a *According to *CYPallele* nomenclature homepage http://www.cypalleles.ki*.

b *Genomic (g.) and cDNA (c.) positions are given in bp*.

c *Selected frequencies of individual ethnicities (AA, African American; Af African; As Asian; Ca Caucasian; Hs, Hispanic; PNG, Papua New Guineans) compiled from dbSNP http://www.ncbi.nlm.nih.gov/SNP and from the literature cited in the text*.

## CYP2B6*6 AND EFAVIRENZ: *IN VIVO*, *EX VIVO*, *IN VITRO*

The most common variant allele in all populations studied to date harbors two amino acid changes, Q172H and K262R, and is termed CYP2B6*6. This haplotype occurs in about 15 to over 60% of individuals, depending on ethnicity (**Table 1**). Although additional variants occur in the promoter and in introns, their functional impact appears to be of limited relevance and will not be further discussed here (Lamba et al., 2003; Hesse et al., 2004; Hofmann et al., 2008).

Since the discovery that CYP2B6 is the major enzyme for efavirenz 8-hydroxylation (Ward et al., 2003), pharmacogenetic studies have linked the Q172H variant to elevated plasma concentrations of efavirenz, indicating decreased enzyme function *in vivo.* This finding has been reproduced manifold in different ethnicities throughout the world (summarized by Telenti and Zanger, 2008; Rakhmanina and van den Anker, 2010). Three CYP2B6 polymorphisms, 15631G>T, 21011T>C, and an intron 3 SNP rs4803419, were also shown to be associated with efavirenz pharmacokinetics at genome wide significance (Holzinger et al., 2012).

The potent first-generation NNRTI of HIV-1 is recommended as initial therapy with two NRTIs in highly active antiretroviral therapy (HAART) regimes, but patients with subtherapeutic plasma concentrations can develop resistance and treatment failure, whereas those with too high plasma levels are at increased risk of central nervous system (CNS) side effects, which can lead to treatment discontinuation in a fraction of patients (King and Aberg, 2008). Q172H variant was furthermore associated with increased neurotoxicity and other CNS side effects (Haas et al., 2004; King and Aberg, 2008; Lubomirov et al., 2010; Ribaudo et al., 2010; Maimbo et al., 2011) with HAART-induced liver injury (Yimer et al., 2011), and with efavirenz treatment discontinuation and the associated risk of developing drug resistance (Ribaudo et al., 2006; Lubomirov et al., 2011; Wyen et al., 2011). Importantly, compound heterozygotes of 516T and another low activity allele (e.g., *11, *18, *27, *28) also predict high efavirenz plasma levels (Rotger et al., 2007; Ribaudo et al., 2010). In prospective, genotype-based dose adjustment studies the therapeutic dose of efavirenz could be successfully reduced and CNS-related side effects decreased (Gatanaga et al., 2007; Gatanaga and Oka, 2009). Using pharmacokinetic modeling and simulation it was suggested that *a priori *dose reduction in homozygous CYP2B6*6 patients would maintain drug exposure within the therapeutic range in this group of patients (Nyakutira et al., 2008).

**FIGURE 2:**
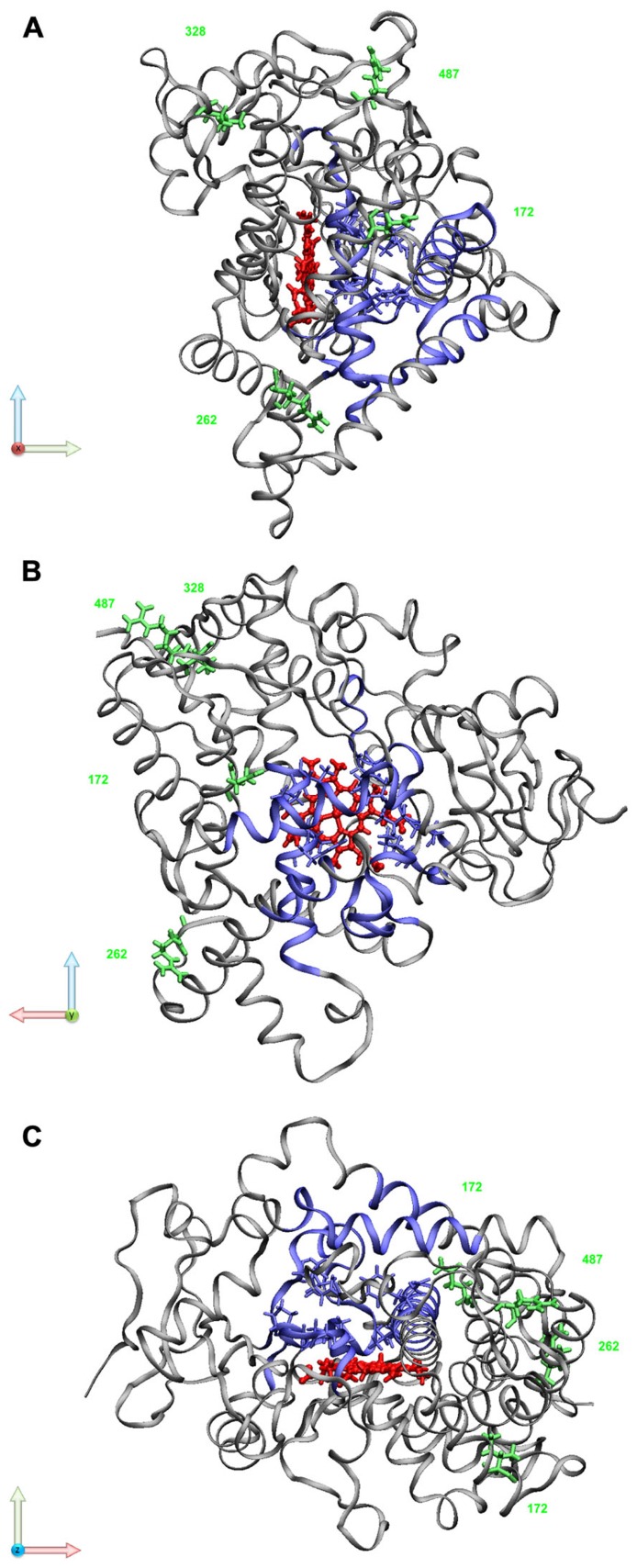
**CYP2B6 structural model**. Selected variant residues are marked in green (Q172H, K262R, I328T, R487C); substrate recognition sites (SRS; Nguyen et al., 2008) and active site residues (ASR; Niu et al., 2011) are highlighted in blue; the prosthetic heme molecule is shown in red. PDB file 3IBD was visualized using VMD visual molecular dynamics viewer http://www.ks.uiuc.edu/Research/vmd/ (Humphrey et al., 1996). The protein sequence used for determining the crystal structure contains R at position 262 instead of the reference residue K. **(A)** view along *x*-axis (light red); **(B)** view along *y*-axis (light green); **(C)** view along *z*-axis (light blue)

The *in vitro *data that have accumulated over the years on the CYP2B6*6 allele draw a more complex picture with functional consequences on various levels including pre-mRNA splicing, protein expression, as well as substrate-dependent changes in enzyme activity and different sensitivity toward irreversible inhibition. While early studies using recombinantly expressed enzyme variants found higher 7-ethoxycoumarin O-deethylase activity for the Q172H variant (Ariyoshi et al., 2001; Jinno et al., 2003), in genotyped human livers (*ex vivo*), the *6 allele has been associated with approximately 50–75% decreased protein levels (Lang et al., 2001; Desta et al., 2007; Hofmann et al., 2008). An explanation for decreased protein expression was provided based on the observation that the c.516G>T SNP coding for Q172H in exon 4 (rs3745274, **Table 1**) was correlated to increased amounts of a hepatic splice variant that lacked exons 4–6, and concurrently to decreased amounts of the normal functional transcript. Recombinant expression of minigene constructs in mammalian cells proved that the c.516G>T variant was causally involved in erroneous splicing and lower expression of functional mRNA and protein (Hofmann et al., 2008). It has been hypothesized that binding of splice factors to an exonic splicing enhancer(s) located in exon 4 could be affected by the variant (Zanger and Hofmann, 2008; Sadee et al., 2011). Although reduced expression in liver appears to satisfactorily explain increased efavirenz plasma concentrations in individuals with *6/*6 genotype (Desta et al., 2007), recent *in vitro *data of expressed variants seem to indicate that the amino acid substitutions contribute to changes in catalytic activity of the enzyme. Structurally this is not easy to comprehend, because the Q172H and Lys262Arg amino acid changes occur in regions of the protein that are not directly located at the active site or that have been identified as substrate recognition sites (**Figure 2)**.

Concerning efavirenz and also other substrates, the available *in vitro* data are however, not well in agreement with each other. **Table 2** summarizes kinetic parameters for bupropion and efavirenz for CYP2B6 enzyme variants obtained from different recombinant expression systems. Using *Escherichia coli *expression system, Zhang et al. (2011b) purified six *N-*terminally truncated expressed variants to homogeneity and reconstituted them with NADPH:cytochrome 450 reductase (POR) at a molar ratio of 1:2 and measured efavirenz and bupropion kinetics. Using Sf9 insect cell cotransfection, CYP2B6.1, 2B6.4 and 2B6.6 were expressed in the presence of 10-fold excess of POR, i.e., under saturating conditions, to measure efavirenz kinetics (Ariyoshi et al., 2011). Another study determined both bupropion and efavirenz kinetics in protein preparations also derived from insect cells in the presence or absence of cytochrome b5 (CYB5) but at somewhat more variable ratios in regard to POR (Xu et al., 2012). Radloff et al. (2013) used the COS-1 expression system, where P450 monooxygenase activity is supported by endogenously expressed POR, to determine bupropion and efavirenz kinetics for several novel CYP2B6 variants in comparison to the known variants 2B6.1, 2B6.5, and 2B6.6.

**Table 2:** Kinetic properties of recombinantly expressed CYP2B6 protein variants with bupropion and efavirenz.

Variant	System	Bupropion hydroxylation			Efavirenz 8-hydroxylation			Reference
		K_m_(μM)	*V*_max_ (%)	CL_int_ (%)	K_m_(μM)	*V*_max_ (%)	CL_int_ (%)
2B6.1	COS-1	87	100	100	2.07	100	100	Radloff et al. (2013)
	*E. coli*	95	100	100	7.3	100	100	Zhang et al. (2011b)
	Sf9				7.7	100	100	Ariyoshi et al. (2011)
	Sf9	64	100	100	3.2	100	100	Xu et al. (2012)
2B6.6	COS-1	72	81	98	1.21	107	183	Radloff et al. (2013)
	*E. coli*	380	175	43	198	563	20	Zhang et al. (2011b)
	Sf9				12.4	81	50	Ariyoshi et al. (2011)
	Sf9	63	139	143	8.8	133	49	Xu et al. (2012)
2B6.4	*E. coli*	162	60	35	5.5	73	96	Zhang et al. (2011b)
	Sf9				9.16	169	142	Ariyoshi et al. (2011)
2B6.5	COS-1	65	44	59	1.15	46	83	Radloff et al. (2013)
	*E. coli*	134	66	47	53	1005	138	Zhang et al. (2011b)

*Only studies which determined kinetic parameters (*K*_m_, *V*_max_, or *K*_cat_) were included.*

The compilation of data in **Table 2** shows that differences between the variants were masked by differences between the expression systems. For example, efavirenz *K*_m_ was moderately decreased (58%) for COS-1 cell-expressed 2B6.6 compared to 2B6.1 but moderately larger for both insect cell-expressed proteins. The *E. coli*-expressed variant showed, however, 27-fold increased *K*_m_. While the COS-1 proteins had almost identical *V*_max_, one of the insect cellproteins had decreased *V*_max_ (81%), while the other had increased activity (133%). Again, the *E. coli*-expressed variant showed the biggest difference of almost sixfold higher activity for the variant. Similar discrepancies, albeit less dramatic, were found with bupropion as substrate (**Table 2**).

This data-comparison illustrates the problems that still exist with recombinant P450 expression systems, and particularly for CYP2B6, which appears to be an enzyme that sensibly reacts with activity changes to expression conditions. It is difficult to pin down the reasons for these differences exactly. Reconstitution of recombinant or even purified P450 with POR and CYB5 is a non-trivial problem especially if different protein variants shall be compared for quantitative kinetic parameters. Reconstitution under saturating conditions with respect to electron donators, e.g., at a POR:P450 ratio of 10, is a straightforward practical way, but in hepatocytes, POR is stoichiometrically underrepresented (ratio about 1:10) and may be limiting for monooxygenase activity (Gomes et al., 2009). Enzyme variants may interact differently with the electron donors and catalytic differences could thus depend on reconstitution conditions. In addition, *N-*terminal modifications required to achieve high expression in *E. coli *may interact with the DNA-polymorphisms to be analyzed. In the COS-cell system, on the other hand, the POR:P450 ratio can neither be controlled nor quantified because expression of P450 is too low for spectral quantitation.

Taken together, the data from expression systems indicated that catalytic differences may exist between CYP2B6.6 and CYP2B6.1. However, except for the *E. coli *study, the differences were rather modest and at present it cannot be concluded with certainty whether the CYP2B6.6 variant is catalytically more or less active compared to the wild-type, atleast for bupropion and efavirenz. Taken all evidence together, the decrease in hepatic expression due to erroneous splicing caused by the c.516G>T SNP (Desta et al., 2007; Hofmann et al., 2008) most plausibly explains most of the phenotypic *in vivo *activity differences observed with efavirenz and bupropion.

CYP2B6*6 SNP-related functional differences were also observed with inhibitors. In contrast to the wild-type enzyme the recombinantly expressed K262R variant was not inactivated by efavirenz, but both enzymes were irreversibly inhibited by 8-hydroxyefavirenz (Bumpus et al., 2006; Bumpus and Hollenberg, 2008). Lower susceptibility to inhibition of the K262R variant and the CYP2B6.6 double variant compared to CYP2B6.1 was also found with respect to sertraline and clopidogrel, as well as several other potent drug inhibitors of CYP2B6 (Talakad et al., 2009). These data indicate a role of genetic polymorphisms in drug–drug interaction sensitivity of CYP2B6, a finding that warrants further investigation *in vivo*.

## OTHER *CYP2B6* VARIANTS AND OTHER SUBSTRATES – *IN VITRO* STUDIES

The two amino acid changes that together constitute the *6 allele also occur in isolation, although at much lower frequencies (Klein et al., 2005; Zanger et al., 2007; **Table 1**). In most pharmacogenetic studies they are not being determined and functional data is therefore rare, especially concerning *9 (Klein et al., 2005)*. *Data from recombinant systems as well as liver data suggest that the K262R variant possesses catalytic activities similar to the wild-type enzyme, although with different substrates moderately increased or decreased activity was observed (**Tables 2** and **3**; Desta et al., 2007; Hofmann et al., 2008). The allele with amino acid change [R487C] in exon 9 (*5 variant; **Table 1**) expresses very low levels of protein which does not translate into similarly reduced activities as measured with bupropion as well as efavirenz (Lang et al., 2001; Desta et al., 2007; Hofmann et al., 2008). The variant was shown to have higher specific activity compared to wild-type (Radloff et al., 2013). In human liver, this leads to partial compensation of low expression, finally resulting in a phenotype with moderately decreased activity with bupropion and efavirenz *in vivo*. This explains why CYP2B6*5 was not associated with efavirenz pharmacokinetics in HIV patients (Burger et al., 2006).

**Table 3:** Properties of recombinantly expressed CYP2B6 protein variants with other clinical substrates.

	Artemether^1^ COS-7 cells (Honda et al., 2011)		Selegiline^2^ COS-7 cells (Watanabe et al., 2010)		Chlorpyrifos^3^ COS-1 cells (Crane et al., 2012)		Cyclophosphamide^4^ Sf9 cells (Ariyoshi et al., 2011)		Cyclophosphamide^4^ *E. coli* (Raccor et al., 2012)	
Variant	*K*_m_ (μM)	*V*_max_ (%)	*K*_m_ (μM)	*V*_max_ (%)	*K*_m_ (μM)	*V*_max_ (%)	*K*_m_ (mM)	*V*_max_ (%)	*K*_m_ (mM)	*V*_max_(%)
2B6.1	3.1	100	48.2	100	1.84	100	2.68	100	3.6	100
2B6.6	6.72	416	56.6	169	1.97	254	1.62	99	4.0	155.2
2B6.4	2.73	196	45.8	147	1.09	1094	2.75	74	3.5	67.1
2B6.5	6.87	55	70.1	85	0.80	441			5.1	72.4

1 *O-Demethylation*.

2 *N-Demethylation (mean values were calculated for several expressions of CYP2B6.1)*.

3 *Desulfation*.

4 *4-Hydroxylation*.

The second most important functionally deficient allele is CYP2B6*18 (c.983C>T [I328T]), which occurs predominantly in African subjects with allele frequencies of 4–11% (Mehlotra et al., 2007; Li et al., 2012). The I328T variant expressed no detectable protein or activity toward bupropion, 7-ethoxy-4-trifluoromethylcoumarin (7-EFC), selegiline and artemether in COS-1 cells whereas a partially defective protein was expressed in insect cells (Klein et al., 2005; Watanabe et al., 2010; Honda et al., 2011). This demonstrates another example for expression system-dependent differences. Most likely the 2B6.18 variant is temperature-sensitive and thus able to be expressed at the lower temperature (27°C) of insect cell culture but not at 37°C. Interestingly, the I328T+Q172H double variant expressed partially functional protein in HEK293 cells and in yeast (Wang et al., 2006), indicating that Q172H can stabilize the I328T variant. The *18 allele is thus phenotypically a null allele, at least *in vitro *with some substrates. This is supported by many *in vivo* studies (see below).

At least 12 additional null or low-activity alleles have been described and analyzed with various substrates (Lang et al., 2004; Klein et al., 2005; Rotger et al., 2007; Watanabe et al., 2010; Honda et al., 2011). Although they are rather rare in all investigated populations they may have profound effects on drug metabolism if present in compound heterozygous genotypes, e.g., in combination with *6 or *18 (Rotger et al., 2007). The CYP2B6*22 allele is a gain-of-function variant associated with increased transcription *in vitro *(Zukunft et al., 2005) and with increased activity *in vivo *(Rotger et al., 2007). It was shown that a -82T>C exchange alters the TATA-box into a functional CCAAT/enhancer-binding protein binding site that causes increased transcription from an alternative downstream initiation site (Zukunft et al., 2005). Interestingly, the -82T>C polymorphism also confers synergistically enhanced *CYP2B6* inducibility by the PXR ligand rifampicin in human primary hepatocytes (Li et al., 2010).

New variants are discovered preferentially in previously uncharacterized ethnic groups. Restrepo et al. (2011) described two novel combinations of known amino acid variants in a Colombian population. Structural variants including a novel CYP2B6/2B7P1 duplicated fusion allele (CYP2B6*30) were found when individuals from various ethnicities were screened for copy number variations (Martis et al., 2012). Furthermore, three novel and five previously uncharacterized amino acid variants in different combinations (CYP2B6*33 to *37) were identified by resequencing the *CYP2B6 *gene in a Rwandese cohort of HIV-1-infected patients (Radloff et al., 2013). The variants were then functionally studied by COS-1 cell expression and by *in silico* prediction tools. At least four of the variants were shown to result in complete or almost complete loss of function with bupropion and efavirenz as substrates. The detailed comparison of *in vitro* functionality of the variants with *in silico* prediction tools including a thorough substrate docking simulation analysis points at the challenge to deal with the hundreds of new variants that exist in all populations as currently uncovered by next generation sequencing approaches and large scale population projects (see links above).

## CLINICAL STUDIES WITH DIFFERENT DRUGS

The widely used anticancer and immunosuppressant prodrug cyclophosphamide depends on bioactivation to 4-hydroxycyclophosphamide for cytotoxic activity. Bioactivation is highly variable in cancer patients and has been attributed mainly to CYP2B6 *in vitro *and *in vivo *with contributions from CYP2C19 and CYP3A4 (Chang et al., 1993; Raccor et al., 2012). The case of cyclophosphamide 4-hydroxylation deserves particular attention, as it exemplifies substrate-dependent effects of CYP2B6 pharmacogenetics. Cyclophosphamide 4-hydroxylation was initially reported to be enhanced in livers genotyped CYP2B6*6/*6 (Xie et al., 2003), which was confirmed in several later *in vivo *studies (Xie et al., 2006; Nakajima et al., 2007; Torimoto and Kohgo, 2008). However, other *in vivo* studies analyzing pharmacokinetics or clinical outcome also presented contradictory or negative results (Singh et al., 2007; Ekhart et al., 2008; Melanson et al., 2010; Yao et al., 2010; Raccor et al., 2012). *In vitro, *insect cell-expressed recombinant CYP2B6.4 [K262R] had lower activity for cyclophosphamide 4-hydroxylation (Ariyoshi et al., 2011; Raccor et al., 2012). The CYP2B6.4 and CYP2B6.6 variants thus display mirror-inverted catalytic activities toward efavirenz and cyclophosphamide, in that the former variant is the catalytically more active one with efavirenz, whereas the opposite is true for the latter variant (**Table 3**). A direct comparison of catalytic properties of the two variants with the reference enzyme expressed in insect cells supports this inverse behavior of the two variants toward these two substrates (Ariyoshi et al., 2011). Interestingly, several studies associated other variants including CYP2B6*4, *5, *8,**and *9 with lower 4-OH cyclophosphamide formation *in vivo *or with worse outcome (Takada et al., 2004; Bray et al., 2010; Helsby et al., 2010; Joy et al., 2012). Taken together, the data concerning cyclophosphamide from both *in vivo* and *in vitro *indicate that *CYP2B6* polymorphism plays a role, although the studies are so far not yet conclusive. This may be explained by different study size design as well as lack of consistency in allele definition and genotype information among studies (Helsby and Tingle, 2011).

In addition to efavirenz, *CYP2B6* genotype also affects plasma levels of the antiretroviral drug nevirapine (Penzak et al., 2007; Mahungu et al., 2009). The impact of the *CYP2B6* 516G>T polymorphism on nevirapine exposure was confirmed and quantified in a pharmacometric analysis of nevirapine plasma concentrations from 271 patients genotyped for 198 SNPs in 45 ADME (absorption, distribution, metabolism, and excretion) genes and covariates (Lehr et al., 2011). Moreover, nevirapine-related cutaneous adverse events, which are most likely major histocompatibility complex (MHC) class I-mediated, were significantly influenced by *CYP2B6 *polymorphism while hepatic side effects, most likely MHC class II-mediated, were unaffected by *CYP2B6* (Yuan et al., 2011).

*CYP2B6 *allele variants were also investigated in the context of the synthetic μ-opioid receptor agonist, methadone, which is metabolized by CYPs 3A4/5, 2B6, and 2D6, and used as a maintenance treatment for opioid addiction. In *6/*6 carriers (*S*)-methadone plasma levels were increased leading to potentially higher risk of severe cardiac arrhythmias and methadone associated deaths (Crettol et al., 2005; Eap et al., 2007; Bunten et al., 2011). Methadone dose requirement for effective treatment of opioid addiction was shown to be significantly reduced in carriers of this genotype (Levran et al., 2011).

## CONCLUSION

The polymorphism of the *CYP2B6* gene has initially been studied by reverse genetics approach, i.e., starting from the identification of genetic variants in DNA and liver samples, followed by *in vitro* characterization of genotyped livers and expressed variant proteins. Clinical studies have then contributed to identify the variants that are important *in vivo*, and *in vitro* studies are again needed to identify and mechanistically explain causal variants. Nevertheless, C*YP2B6* pharmacogenetics has yet to be fully explored, especially with respect to combined effects of the involved variants on both expression and catalytic properties, the latter of which additionally depend on the substrate. While the relevance for HIV-1 therapy with efavirenz is well established and translational approaches have already been clinically tested, an increasing number of studies suggest clinical relevance for additional drug substrates.

## Conflict of Interest Statement

Ulrich M. Zanger is a named coinventor of a patent on the detection of specific CYP2B6 polymorphisms for diagnostic purposes. Kathrin Klein declares no conflict of interest.

## Footnotes

1 http://www.cypalleles.ki.se/cyp2b6.htm

2 http://www.pharmgkb.org/

3 http://www.ncbi.nlm.nih.gov/projects/SNP/

4 http://www.1000genomes.org/

5 http://EVS.gs.washington.edu
